# The Relationship of Alcohol Consumption and Drinking Pattern to the Risk of Glomerular Hyperfiltration in Middle-aged Japanese Men: The Kansai Healthcare Study

**DOI:** 10.2188/jea.JE20220312

**Published:** 2024-03-05

**Authors:** Mikiko Shibata, Kyoko Kogawa Sato, Hideo Koh, Izumi Shibata, Kaori Okamura, Yuka Takeuchi, Keiko Oue, Michio Morimoto, Tomoshige Hayashi

**Affiliations:** 1 Preventive Medicine and Environmental Health, Osaka Metropolitan University Graduate School of Medicine, Osaka, Japan; 2 Preventive Medicine and Environmental Health, Osaka City University Graduate School of Medicine, Osaka, Japan; 3 Health Administration Center (Kansai region), Nippon Telegraph and Telephone West Corporation, Osaka, Japan

**Keywords:** alcohol, drinking pattern, glomerular hyperfiltration, prospective cohort study, epidemiology

## Abstract

**Background:**

Glomerular hyperfiltration has been reported to be associated with adverse renal outcomes in the general population. It is not known whether drinking pattern is associated with the risk of glomerular hyperfiltration in healthy individuals.

**Methods:**

We prospectively followed middle-aged 8,640 Japanese men with normal renal function, no proteinuria, no diabetes, and no use of antihypertensive medications at entry. Data on alcohol consumption were gathered by questionnaire. Glomerular hyperfiltration was defined as estimated glomerular filtration rate (eGFR) ≥117 mL/min/1.73 m^2^, which was the upper 2.5th percentile value of eGFR in the entire cohort.

**Results:**

During 46,186 person-years of follow-up, 330 men developed glomerular hyperfiltration. In a multivariate model, for men who consumed alcohol on 1–3 days per week, alcohol consumption of ≥69.1 g ethanol/drinking day was significantly associated with the risk of glomerular hyperfiltration (hazard ratio [HR] 2.37; 95% confidence interval [CI], 1.18–4.74) compared with non-drinkers. For those who consumed alcohol on 4–7 days per week, higher alcohol consumption per drinking day was associated with a higher risk of glomerular hyperfiltration: the HRs for alcohol consumption of 46.1–69.0, and ≥69.1 g ethanol/drinking day were 1.55 (95% CI, 1.01–2.38), and 1.78 (95% CI, 1.02–3.12), respectively.

**Conclusion:**

For high drinking frequency per week, more alcohol intake per drinking day was associated with an increased risk of glomerular hyperfiltration, while for low drinking frequency per week, only very high alcohol intake per drinking day was associated with an increased risk of glomerular hyperfiltration in middle-aged Japanese men.

## INTRODUCTION

Glomerular hyperfiltration, an abnormally high glomerular filtration rate (GFR), is a well-known phenomenon found early in the clinical course of diabetes^1^^,^^2^ and has been reported to be associated with an increased risk of subsequent diabetic nephropathy^3^ and rapid renal decline.^4^^–^^8^ Even in the general population, glomerular hyperfiltration is an important issue that needs attention because relationships of glomerular hyperfiltration to subsequent renal decline,^9^ cardiovascular disease,^10^ and all-cause mortality^11^ have been reported. To prevent or delay the onset of these serious events, it is critical to examine the risk of developing glomerular hyperfiltration. Prediabetes and lifestyle factors, such as obesity,^12^^,^^13^ cigarette smoking,^14^ and high protein diets,^15^^,^^16^ have been reported as risk factors for glomerular hyperfiltration, but it has not yet been fully investigated whether drinking habits are associated with the risk of glomerular hyperfiltration.

Light to moderate alcohol consumption has been reported to have beneficial effects on the incidence of type 2 diabetes^17^ and coronary heart disease.^18^ Although, in many of the epidemiological studies included in these meta-analyses, drinking habits have often been assessed by weekly or average daily alcohol consumption, this method fails to distinguish between the contribution of drinking and non-drinking days. Drinking patterns represent drinking habits that take into account the frequency of drinking and the amount of alcohol consumed per drinking day. These drinking patterns also have crucial impacts on the risk of those diseases.^19^^–^^22^ In our previous study, light alcohol consumption per drinking day (0.1–23.0 g ethanol per drinking day) on 4–7 days per week was significantly associated with a decreased risk of developing proteinuria compared to nondrinkers, while heavy alcohol consumption per drinking day (≥69.1 g ethanol/drinking day) on 1–3 days per week was significantly associated with an increased risk of developing proteinuria.^19^ Previous epidemiological studies have reported that subjects with similar weekly alcohol intake (similar average daily alcohol intake) have different risks of coronary heart disease^20^ and prostate cancer^21^ if they drink at different frequencies. In the Health Professionals Follow-up Study, increasing frequency of drinking per week was associated with a decreased risk of type 2 diabetes, and among those who drank 5–7 days per week, those who drank small amounts per drinking day had the lowest risk of type 2 diabetes.^22^ Therefore, when examining the relationship between drinking habits and risk of disease, it is more important to simultaneously consider both the frequency of drinking and the amount of alcohol consumed per drinking day than to consider average daily alcohol consumption. However, both weekly or average daily alcohol consumption and drinking patterns have not been fully examined in relation to the risk of glomerular hyperfiltration. Only one cross-sectional study^23^ and one prospective study^14^ were available on the association between average alcohol consumption and glomerular hyperfiltration. We previously reported a positive association between average daily alcohol consumption as a continuous covariate and the incidence of glomerular hyperfiltration in men participating in the Kansai Healthcare Study but have not examined this relationship in detail.^14^ To our knowledge, no prospective data are available regarding the relationship of drinking pattern to the development of glomerular hyperfiltration.

Therefore, we prospectively investigated the relationships of detailed daily average alcohol consumption and drinking pattern to the risk of developing glomerular hyperfiltration during the 6-year observation period in middle-aged Japanese men.

## METHODS

### Site and setting

The Kansai Healthcare Study, a prospective cohort study, was designed to investigate risk factors for cardiometabolic diseases. A detailed description of the study has been presented elsewhere.^24^ Between April 2000 and March 2001, we enrolled 12,647 male employees of a company in the area of Kansai, Japan, who were 40 to 55 years old at entry and considered to be involved in sedentary jobs. All employees in the company over 40 years of age are required by Japanese law to undergo annual medical examinations. The research protocol received approval from the ethics committee of Osaka City University Graduate School of Medicine.

Participants were eligible for the present analysis if they had normal renal function and no proteinuria at entry. The current investigation is consisted of 11,268 men whose baseline estimated glomerular filtration rate (eGFR) was ≥60 mL/min/1.73 m^2^ and <117 mL/min/1.73 m^2^, and who had no proteinuria using the urine dipstick test. We excluded 1,458 men who had a fasting plasma glucose (FPG) ≥126 mg/dL, or who were taking glucose-lowering agents, insulin, or antihypertensive drugs at entry. We also excluded 352 men because of the missing covariates and 364 men who never had an annual medical examination during the follow-up period. Furthermore, 454 men whose eGFR decreased to less than 60 mL/min/1.73 m^2^ during the follow-up period were also eliminated from the current analysis. After exclusions, 8,640 men remained eligible.

### Data collection and measurements

The data collected in the study were medical history; lifestyle characteristics, such as smoking habits, regular leisure-time physical activity, and alcohol drinking habits; anthropometric measurements; blood sampling for the FPG and serum creatinine; and urine sample for dipstick urinalysis. Trained nurses collected all measurements. Blood samples were taken after over-night 12-hour fasting. In most participants, the level of serum creatinine was determined using an enzymatic method on a Hitachi 7350 automatic chemistry analyzer (Hitachi Ltd., Tokyo, Japan). In 1,557 participants, the level of serum creatinine was determined using the Jaffe method. We recalibrated the Jaffe values to enzymatic values using the equation that has been described elsewhere in detail.^19^ Then eGFR was calculated utilizing the Modification of Diet in Renal Disease study equation for Japanese men as follows: eGFR = 194 × age^−0.287^ × serum creatinine^−1.094^ (mg/dL, enzymatic method).^25^ Dipstick urinalysis was performed on urine samples collected as midstream, clean-catch, and random urine specimens. The urinalysis results were interpreted as negative, ±, 1+, 2+, 3+, or 4+. The results of ± or less were considered as normal. Height and weight were measured, and body mass index (BMI) was computed by taking the weight in kilograms divided by the height in meters squared. Blood pressure was measured on the right arm of seated participants after 5 minutes of rest with an automated sphygmomanometer (BP-203RV; Omron Colin, Tokyo, Japan, and Udex-super; ELK, Osaka, Japan). Hypertension at baseline was defined as systolic blood pressure ≥140 mm Hg or diastolic blood pressure ≥90 mm Hg.^26^ According to the American Diabetes Association criteria,^27^ participants at baseline were classified as normal fasting glucose (FPG <100 mg/dL), impaired fasting glucose (FPG 100–125 mg/dL), or diabetes (FPG ≥126 mg/dL).

The participants completed self-administered questionnaires on alcohol drinking habits, smoking habits, and regular leisure-time physical activity. For smoking habits, the study participants were grouped into three categories: those who have never smoked, those who used to smoke, and those who currently smoke. We asked a question about regular leisure-time physical activity with the following three options: rarely, sometimes, and regular (that is, at least once weekly). The participants were divided into two groups based on their leisure-time physical activity frequency: those who engaged in physical activity at least once a week and those who engaged in physical activity less than once a week.^24^

### Measurement of alcohol consumption

In the questionnaire on alcohol consumption, the frequency of drinking per week (how many days per week they drink) and the amount of alcohol consumed per day were evaluated. The alcohol content of Japanese sake is generally 15–16%,^28^ and 1 gou of sake (180 mL) corresponds to 22–23 g of ethanol. We used 23 g of ethanol as the basis for the calculation. Average daily alcohol consumption was calculated as follows (the amount of alcohol consumed per drinking day) × (the number of drinking days per week)/7. Based on their average daily alcohol consumption, we classified the participants into the following categories: non-drinkers, 0.1–23.0 g ethanol/day, 23.1–46.0 g ethanol/day, 46.1–69.0 g ethanol/day, and ≥69.1 g ethanol/day.

Drinking pattern was determined by combining the number of drinking days per week and the amount of alcohol consumed per drinking day. For the number of drinking days per week, participants were categorized as “non-drinkers”, “drinkers 1–3 days per week”, and “drinkers 4–7 days per week”. Regarding the amount of alcohol consumed per drinking day, the participants were classified as non-drinkers, those who consumed 0.1–23.0 g of ethanol, those who consumed 23.1–46.0 g of ethanol, those who consumed 46.1–69.0 g of ethanol, and those who consumed ≥69.1 g of ethanol.

### Outcomes

The study outcome was the incidence of glomerular hyperfiltration. Glomerular hyperfiltration was defined as eGFR ≥117 mL/min/1.73 m^2^ using the data of annual medical examinations during the follow-up period. This threshold was defined as the upper 2.5th percentile value of baseline eGFR in the entire cohort.

### Statistical analyses

We used multivariate Cox proportional hazards models to examine the relationship of average daily alcohol consumption and drinking pattern at baseline to the incidence of glomerular hyperfiltration. Follow-up of each participant was continued either until the outcome occurrence or until the 6-year follow-up examination from April 1, 2006 to March 31, 2007, whichever occurred first. We assessed the nonlinear effects of continuous independent variables using fractional polynomials.^29^ We also assessed these effects using quadratic, square root, and log transformations. In multivariate models using Cox proportional hazards models, as the relationship of FPG with the risk of glomerular hyperfiltration was nonlinear, we fitted models using the categorized variable of FPG: <100 mg/dL, and 100–125 mg/dL.^27^ The assumption of proportional hazards for covariates was assessed by inserting time-dependent covariates or using Schoenfeld residuals plot and Schoenfeld residuals test.^30^ The presence of effect modification was tested by the insertion of first-order interaction terms between BMI and average daily alcohol consumption or BMI and drinking pattern into appropriate models. Although we assumed that an important factor explaining the relationship between drinking habits and the development of glomerular hyperfiltration is the involvement of insulin resistance, a measure of insulin resistance was not measured in this study. Therefore, we used BMI as an alternative index of insulin resistance and examined the effect modification by BMI. Multicollinearity was assessed using the variance inflation factor.^31^ Outliers were identified by plotting the likelihood displacement and LMAX values of all independent variables.^30^ No outliers were detected in any of the models. Tests for trends were conducted by assigning the median value to each category of drinking pattern and modeling this value as a continuous variable. The 95% confidence interval (CI) for each hazard ratio (HR) was calculated. All reported *P* values were two-sided. Statistical analysis was performed using Stata MP, Version 17.0 (Stata Corp., College Station, TX, USA).

## RESULTS

### Baseline characteristics

Baseline characteristics of participants according to average daily alcohol consumption are shown in Table 1. Participants who consumed higher levels of alcohol per day tended to have higher systolic blood pressure, diastolic blood pressure, FPG, and eGFR, as well as a higher proportion of hypertension.

**Table 1.** Baseline characteristics according to average daily alcohol consumption

	Total	Average daily alcohol consumption (g ethanol/day)				

		Non-drinkers	0.1–23.0	23.1–46.0	46.1–69.0	≥69.1
*n*	8,640	1,275	3,628	2,748	805	184
Age, years	48.2 (4.2)	48.8 (4.2)	47.9 (4.2)	48.3 (4.1)	48.1 (4.2)	48.0 (4.2)
Body mass index, kg/m^2^	23.2 (2.8)	23.1 (3.0)	23.3 (2.8)	23.3 (2.8)	23.2 (3.0)	23.1 (3.2)
Systolic blood pressure, mm Hg	127.4 (17.6)	122.8 (17.2)	125.9 (17.1)	129.9 (17.5)	131.5 (17.9)	135.1 (19.0)
Diastolic blood pressure, mm Hg	79.5 (11.8)	76.0 (11.3)	78.7 (11.8)	81.2 (11.6)	81.7 (11.6)	84.9 (12.4)
Hypertension, *n* (%)	2,397 (27.7)	234 (18.4)	911 (25.1)	882 (32.1)	285 (35.4)	85 (46.2)
Fasting plasma glucose, mg/dL	97.2 (9.2)	95.7 (9.0)	97.0 (9.0)	97.8 (9.5)	98.3 (9.5)	98.6 (9.3)
Serum creatinine, mg/dL	0.79 (0.10)	0.79 (0.10)	0.79 (0.10)	0.78 (0.10)	0.77 (0.10)	0.75 (0.10)
Estimated glomerular filtration rate, mL/min/1.73 m^2^	84.6 (12.0)	83.5 (12.2)	83.8 (11.8)	85.5 (11.9)	86.3 (12.2)	88.7 (12.2)
Regular leisure-time physical activity, *n* (%)	1,534 (17.8)	177 (13.9)	652 (18.0)	524 (19.1)	153 (19.0)	28 (15.2)
Smoking status, *n* (%)						
Non-smokers	1,798 (20.8)	297 (23.3)	936 (25.8)	472 (17.2)	79 (9.8)	14 (7.6)
Past smokers	1,859 (21.5)	260 (20.4)	811 (22.3)	605 (22.0)	145 (18.0)	38 (20.7)
Current smokers	4,983 (57.7)	718 (56.3)	1,881 (51.9)	1,671 (60.8)	581 (72.2)	132 (71.7)

Values are expressed as *n* (%) or mean (standard deviation).

### Average daily alcohol consumption and the risk of glomerular hyperfiltration

During the 46,186 person-year period of observation, 330 incident cases of glomerular hyperfiltration were confirmed. Table 2 displays incidence rates and multiple-adjusted hazards ratios about the association between average daily alcohol consumption and the risk of glomerular hyperfiltration. To examine whether there was an effect modification, the first-order interaction term between BMI and average daily alcohol consumption was examined. No significant interaction term was detected. We evaluated multicollinearity using the variance inflation factor, but no multicollinearity was confirmed. In a crude model, participants who consumed 46.1–69.0 g ethanol/day and ≥69.1 g ethanol/day had a significantly higher risk of glomerular hyperfiltration than non-drinkers. After adjustment for age, BMI, systolic blood pressure, smoking status (non-smoker, past smoker, and current smoker), regular leisure-time physical activity (yes/no), and FPG (<100 mg/dL, and 100–125 mg/dL), the same trend was observed but the results were not significant for participants who consumed ≥69.1 g ethanol/day.

**Table 2.** Incidence rates and hazard ratios of incident glomerular hyperfiltration for average daily alcohol consumption

	Incidence rate (cases/person-years)	Crude hazard ratio (95% CI)	*P*-value	Multiple-adjusted hazard ratio^a^ (95% CI)	*P*-value
Average daily alcohol consumption					
Non-drinkers	6.3 (43/6,865)	1.00 (reference)		1.00 (reference)	
0.1–23.0 g ethanol/day	5.3 (104/19,574)	0.85 (0.59–1.21)	0.366	0.87 (0.61–1.24)	0.431
23.1–46.0 g ethanol/day	8.3 (121/14,595)	1.33 (0.94–1.88)	0.110	1.23 (0.87–1.75)	0.247
46.1–69.0 g ethanol/day	12.0 (50/4,153)	1.93 (1.28–2.90)	0.002	1.63 (1.08–2.48)	0.021
≥69.1 g ethanol/day	12.0 (12/999)	1.90 (1.00–3.61)	0.049	1.56 (0.82–2.98)	0.179
*P* for trend^b^		<0.001		<0.001	

CI, confidence interval.

^a^Adjusted for age, body mass index, systolic blood pressure, smoking status (non-smokers, past smokers, and current smokers), regular leisure-time physical activity (yes/no), and fasting plasma glucose (<100 mg/dL and 100–125 mg/dL).

^b^To examine the dose-response relationship between average daily alcohol consumption and the risk of glomerular hyperfiltration, linear trends in risk were examined.

### Drinking pattern and the risk of glomerular hyperfiltration

We examined the joint relationship of the number of drinking days per week and the amount of alcohol consumed per drinking day with the risk of glomerular hyperfiltration (Table 3). Participants were grouped into 9 drinking patterns, which were determined by combining the number of drinking days per week (non-drinkers, 1–3 days/week, 4–7 days/week) and the amount of alcohol consumed per drinking day (0.1–23.0, 23.1–46.0, 46.1–69.0, and ≥69.1 g ethanol/drinking day). The analysis showed an association between drinking pattern and glomerular hyperfiltration. We examined the significance of the first-order interaction term between BMI and drinking pattern. The interaction term was not detected significantly. No multicollinearity was confirmed. In multiple-adjusted model after adjustment for age, BMI, systolic blood pressure, smoking status (non-smokers, past smokers, and current smokers), regular leisure-time physical activity (yes/no), and FPG (<100 mg/dL and 100–125 mg/dL), as for participants who consumed alcohol on 1–3 days per week, only those who consumed ≥69.1 g ethanol/drinking day had a significantly increased risk of glomerular hyperfiltration (HR 2.37; 95% CI, 1.18–4.74) compared with non-drinkers. In contrast, as for participants who consumed alcohol on 4–7 days per week, higher amount of alcohol consumed per drinking day was associated with a higher risk of glomerular hyperfiltration. The multiple-adjusted HRs of incident glomerular hyperfiltration were 1.55 (95% CI, 1.01–2.38) for alcohol consumption of 46.1–69.0 g ethanol/drinking day, and 1.78 (95% CI, 1.02–3.12) for alcohol consumption of ≥69.1 g ethanol/drinking day, compared with non-drinkers. Furthermore, to examine the dose-response relationship between the amount of alcohol consumed on drinking days and the risk of glomerular hyperfiltration, linear trends in risk were examined. For the group that drank 1–3 days per week (with nondrinkers as the reference category), the dose-response trend between the amount of alcohol consumed on drinking days and the risk of glomerular hyperfiltration was not significant. In contrast, for the group that drank 4–7 days per week (non-drinkers as the reference category), the trend in dose-response between the amount of alcohol consumed on drinking days and the risk of glomerular hyperfiltration was significant.

**Table 3.** Incidence rates and hazard ratios of incident glomerular hyperfiltration in relation to drinking pattern

	Incidence rate (cases/person-years)	Crude hazard ratio (95% CI)	*P*-value	Multiple-adjusted hazard ratio^a^ (95% CI)	*P*-value
Non-drinkers	6.3 (43/6,865)	1.00 (reference)		1.00 (reference)	
1–3 drinking days/week					
0.1–23.0 g ethanol/drinking day	5.8 (35/6,059)	0.93 (0.59–1.45)	0.744	0.99 (0.63–1.54)	0.952
23.1–46.0 g ethanol/drinking day	5.8 (29/5,019)	0.92 (0.57–1.47)	0.728	0.93 (0.58–1.49)	0.751
46.1–69.0 g ethanol/drinking day	5.1 (9/1,773)	0.83 (0.40–1.70)	0.607	0.83 (0.41–1.72)	0.625
≥69.1 g ethanol/drinking day	15.8 (10/632)	2.54 (1.28–5.06)	0.008	2.37 (1.18–4.74)	0.015
*P* for trend^b^		0.634		0.705	
4–7 drinking days/week					
0.1–23.0 g ethanol/drinking day	5.0 (40/7,988)	0.80 (0.52–1.23)	0.301	0.79 (0.52–1.22)	0.297
23.1–46.0 g ethanol/drinking day	8.0 (102/12,698)	1.28 (0.90–1.83)	0.168	1.18 (0.82–1.70)	0.368
46.1–69.0 g ethanol/drinking day	11.6 (44/3,781)	1.87 (1.23–2.84)	0.004	1.55 (1.01–2.38)	0.046
≥69.1 g ethanol/drinking day	13.1 (18/1,371)	2.08 (1.20–3.60)	0.009	1.78 (1.02–3.12)	0.042
*P* for trend^c^		<0.001		0.007	

CI, confidence interval.

^a^Adjusted for age, body mass index, systolic blood pressure, smoking status (non-smokers, past smokers, and current smokers), regular leisure-time physical activity (yes/no), and fasting plasma glucose (<100 mg/dL and 100–125 mg/dL).

^b^In a combined subjects of nondrinkers and those who drink 1–3 days per week, to examine the dose-response relationship between the amount of alcohol consumed on drinking days and the risk of glomerular hyperfiltration, linear trends in risk were examined.

^c^In a combined subjects of nondrinkers and those who drink 4–7 days per week, to examine the dose-response relationship between the amount of alcohol consumed on drinking days and the risk of glomerular hyperfiltration, linear trends in risk were examined.

## DISCUSSION

Our prospective data demonstrated that average daily alcohol consumption of 46.1–69.0 g ethanol/day showed a significant association with the risk of incident glomerular hyperfiltration in middle-aged Japanese men. As for drinking pattern, those who consumed ≥69.1 g ethanol per drinking day on 1–3 drinking days per week had the highest risk of developing glomerular hyperfiltration. For those who consumed alcohol on 4–7 days per week, higher alcohol consumption per drinking day was associated with a higher risk of glomerular hyperfiltration compared with non-drinkers. These associations were independent of age, BMI, systolic blood pressure, smoking status, regular leisure-time physical activity, and FPG.

Only one cross-sectional study^23^ and one prospective study^14^ that have related weekly or average daily alcohol consumption to glomerular hyperfiltration. Lin et al have reported in a population-based cross-sectional study in China that alcohol consumption of 210–419 g ethanol per week and ≥420 g ethanol per week were associated with glomerular hyperfiltration, compared with non-drinkers (multiple-adjusted odds ratios 2.45; 95% CI, 1.30–4.61 and 2.67; 95% CI, 1.50–4.77, respectively).^23^ These drinking amounts were equivalent to average daily alcohol consumption of 30–59 g ethanol/day, and ≥60 g ethanol/day, respectively. Because the data in this study were cross-sectional, it was not possible to draw any conclusions about causal relationships. In our previous study examining the association between cigarette smoking and the incident glomerular hyperfiltration, we have reported that average daily alcohol consumption as a continuous variable was significantly associated with the incident glomerular hyperfiltration in a multivariable-adjusted model.^14^ However, our previous study has not examined the relationship of detailed average daily alcohol consumption and drinking pattern to the risk of incident glomerular hyperfiltration.

Drinking habits are assessed using weekly or average daily alcohol intake and drinking patterns. The former method does not accurately distinguish between those who drink small amounts of alcohol daily and those who drink few days per week but drink heavily on drinking days and may place both in the same category. Drinking patterns are drinking habits expressed in terms of frequency of drinking per week and quantity of alcohol consumed per drinking day. Drinking patterns have been shown to have an important relationship with the risk of developing type 2 diabetes, coronary heart disease, and cancer.^20^^–^^22^^,^^24^ The present study showed that drinking patterns are more important than weekly or average daily alcohol intake in relation to the risk of developing glomerular hyperfiltration. Participants who drank ≥69.1 g ethanol per drinking day on 1–3 drinking days per week were at the highest risk of developing glomerular hyperfiltration (Table 3). Their average daily alcohol intake ranged from 5.8 to 41.1 g ethanol/day (eTable 1), which was similar to the average daily alcohol intake of those who drank 0.1 to 46.0 g ethanol per drinking day on 4–7 drinking days per week. However, the multivariate-adjusted HRs for glomerular hyperfiltration for the latter ranged from 0.79 (95% CI, 0.52–1.22) to 1.18 (95% CI, 0.82–1.70), quite different from that for the former (2.37; 95% CI, 1.18–4.74) (Table 3). This indicates that drinking pattern rather than weekly or average daily alcohol consumption is more important for the risk of developing glomerular hyperfiltration. To our knowledge, the present study is the first prospective cohort study to examine the relationship between drinking pattern and the risk of glomerular hyperfiltration.

The mechanisms underlying the association of alcohol consumption with the development of glomerular hyperfiltration are not fully understood, but experimental studies have shown that there are at least three potential mechanisms. First, the activation of the sympathetic nervous system, such as increases in plasma adrenaline^32^ and noradrenaline,^33^ after alcohol consumption have been reported in humans. It has been also reported that glomerular hyperfiltration was evident during sympathetic nervous system activation.^34^ Second, as for the renin-angiotensin-aldosterone system, which has been linked to glomerular hyperfiltration in various conditions such as diabetes and pregnancy,^35^ an experimental study has shown that moderate acute alcohol consumption was related to an elevation in plasma renin activity.^36^ Another potential mechanism may be due to insulin resistance.^37^ It has been reported that the lowest level of the fasting insulin resistance index was found among moderate drinkers, while those with heavy drinking had a higher insulin resistance index.^37^ Furthermore, a recent prospective cohort study has reported that alcohol consumption was positively associated with the incidence of insulin resistance.^38^ Higher insulin resistance has been reported to be related to an increased GFR by ^99m^Tc-diethylenetriaminepentaacetic acid technique in subjects without diabetes and renal diseases.^39^

Regarding the relationship between drinking pattern and glomerular hyperfiltration, the alcohol metabolizing enzyme cytochrome P450 2E1 (CYP2E1) may be involved. Ethanol is primarily metabolized by alcohol dehydrogenase, but when ethanol concentrations are high, CYP2E1 also metabolizes ethanol into acetaldehyde and produces reactive oxygen species (ROS).^40^ Furthermore, chronic alcohol consumption also leads to the upregulation of hepatic CYP2E1 levels, thereby promoting ROS production,^40^ which has been associated with sympathetic nervous system activation,^41^ renin-angiotensin-aldosterone system stimulation,^42^ and insulin resistance.^43^ This may partially explain the association we have shown here between drinking pattern and the development of glomerular hyperfiltration.

There are several limitations to this study. First, since this study was limited to middle-aged Japanese men, it is unclear whether the results are applicable to women, older men, or other ethnic groups. Second, the results might be biased because GFR was not measured directly. Direct measurement of GFR for such a large number of participants is not feasible and this limitation is common in large cohort studies. Estimation of renal function by the Modification of Diet in Renal Disease formula should be permitted.^44^ Third, we did not accumulate more detailed information on alcohol consumption, such as types of beverages and changes in drinking habits during the follow-up period. Therefore, we could not evaluate its relationship with the development of glomerular hyperfiltration. Finally, we could not adjust our models for unknown or unmeasured potential confounders, such as protein intake, which may be important for the development of glomerular hyperfiltration.^15^^,^^16^ Other potential confounders should be included for further research.

In conclusion, our study demonstrated that middle-aged Japanese men with average alcohol consumption of 46.1–69.0 g ethanol/day had a higher risk of glomerular hyperfiltration compared with non-drinkers. According to drinking pattern, men with alcohol consumption of ≥69.1 g ethanol/drinking day on 1–3 days per week had the highest risk of incident glomerular hyperfiltration. Those with higher level of alcohol consumption per drinking day on 4–7 days per week had an increased risk of incident glomerular hyperfiltration. Our findings from this prospective data showed that it is important to reduce the amount of alcohol consumed per drinking day in order to prevent the incidence of glomerular hyperfiltration.

## SUPPLEMENTARY MATERIAL

eTable 1. A detailed description of average daily alcohol consumption according to drinking pattern
