# Supplementary material for: The Relationship of Alcohol Consumption and Drinking Pattern to the Risk of Glomerular Hyperfiltration in Middle-aged Japanese Men: The Kansai Healthcare Study
Source: J Epidemiol. 2024 Mar 5;34(3):137–43. doi: 10.2188/jea.JE20220312 (PMC10853046; doi:10.2188/jea.JE20220312)
Supplement: Supplementary file 1 [file je-34-137-s001.pdf]

eTable 1. A detailed description of average daily alcohol consumption according to drinking pattern

		Average daily alcohol consumption (g ethanol/day)		
Drinking pattern	n	Minimum	Maximum	Median
Non-drinkers	1,275	0	0	0
1–3 drinking days/week				
0.1–23.0 g ethanol/drinking day	1,128	1.6	8.2	1.6
23.1–46.0 g ethanol/drinking day	945	3.3	16.4	16.4
46.1–69.0 g ethanol/drinking day	352	4.9	24.6	24.6
≥69.1 g ethanol/drinking day	125	5.8	41.1	32.9
4–7 drinking days/week				
0.1–23.0 g ethanol/drinking day	1,454	14.8	23.0	23.0
23.1–46.0 g ethanol/drinking day	2,372	29.6	46.0	46.0
46.1–69.0 g ethanol/drinking day	730	49.3	69.0	69.0
≥69.1 g ethanol/drinking day	259	51.8	115.0	74.8
